# 3D quantification of hemodynamics parameters of pulmonary artery and aorta using finite-element interpolations in 4D flow MR data

**DOI:** 10.1186/1532-429X-17-S1-Q27

**Published:** 2015-02-03

**Authors:** Julio A Sotelo, Jesus Urbina, Israel Valverde, Cristian Tejos, Pablo Irarrazaval, Daniel E Hurtado, Sergio Uribe

**Affiliations:** 1 Biomedical Imaging Center, Pontificia Universidad Católica de Chile, Santiago, Chile; 2 Electrical Engineering, Pontificia Universidad Catolica de Chile, Santiago, Chile; 3 Radiology, School of Medicine, Pontificia Universidad Catolica de Chile, Santiago, Chile; 4 Pediatric Cardiology Unit, Hospital Virgen del Rocio, Seville, Spain; 5 Cardiovascular Pathology Unit, Institute of Biomedicine of Seville (IBIS), Seville, Spain; 6 Structural and Geotechnical Engineering, Pontificia Universidad Catolica de Chile, Santiago, Chile; 7 Biomedical Engineering Group, Pontificia Universidad Catolica de Chile, Santiago, Chile

## Background

Several hemodynamic parameters based from 3D cine PC-MRI have been proposed during the last years, including wall shear stress (WSS), oscillatory index (OSI), vorticity and helicity among others. Most of these parameters are quantified using 2D planes, and only few methods have exploited the advantage of using the 3D data. The disadvantage of using 2D planes is that it does not provide the whole distribution of the hemodynamics parameter in the entire vessel of interest. This process is therefore dependent on the user and may lead to results that have low reproducibility. We have developed a computational framework that integrates advanced image processing strategies and computational techniques based on finite element interpolations to perform a 3D quantification of hemodynamics parameters based on 3D cine PC-MRI.

## Methods

To validate the proposed methodology, we acquire data sets in controlled experiments using a realistic aortic phantom normal and with coarctation (Matrix 224x224x122, voxel size 0.89x0.89x0.89 mm, TR/TE 6.3/3.5 ms, Flip angle 100, VENC 250 cm/s). For the phantom experiments we use a MR compatible pulsatile flow pump, which allowed us to resemble the same conditions of velocity and cardiac output as in volunteers and patient with Aortic Coarctation. To demonstrate the applicability of the developed methods, we apply the method in the pulmonary artery and aorta of sixteen volunteers 12 males and 4 females, mean age 30 ± 5 years old (Matrix 106x106x54, voxel size 2.16x2.16x2.48 mm, TR/TE 4.8/2.7 ms, Flip angle 50, VENC 200 cm/s), in which we acquired 3D cine PC-MRI data. Using these data sets we calculate the 3D distribution of WSS, OSI, vorticity and helicity (helicity density and relativity helicity).

## Results

**Figure 1:**
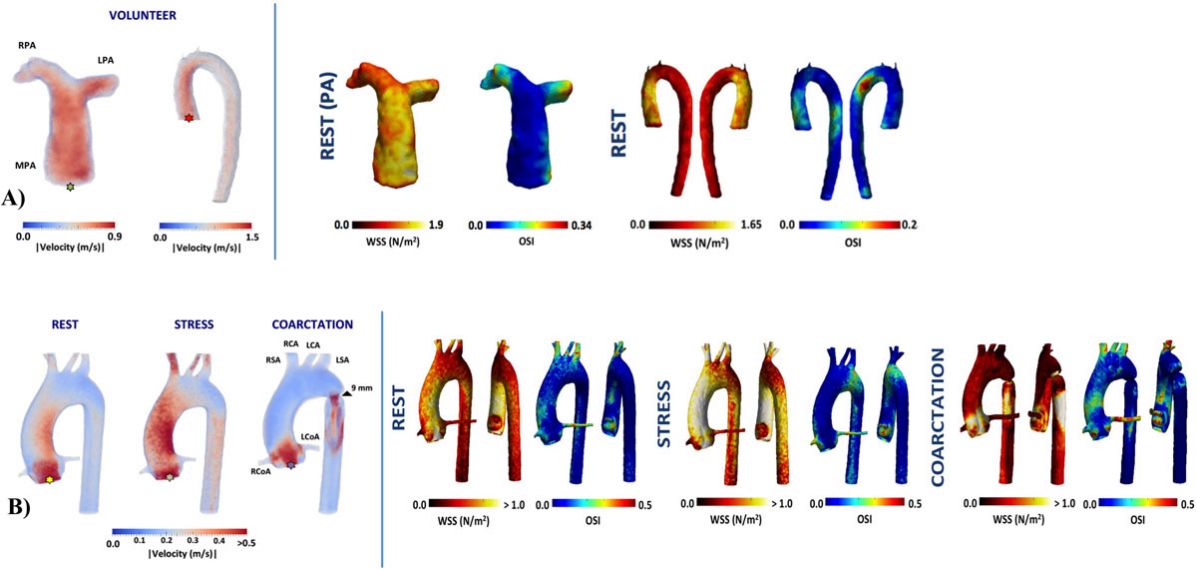
A) 3D velocity magnitude (Left) and 3D maps of wall shear stress magnitud and oscillatory index (Right) of the pulmonary artery and aorta of one volunteer. B) 3D velocity magnitude (Left) and 3D maps of wall shear stress magnitud and oscillatory index (Right) for the phantom data.

**Figure 2:**
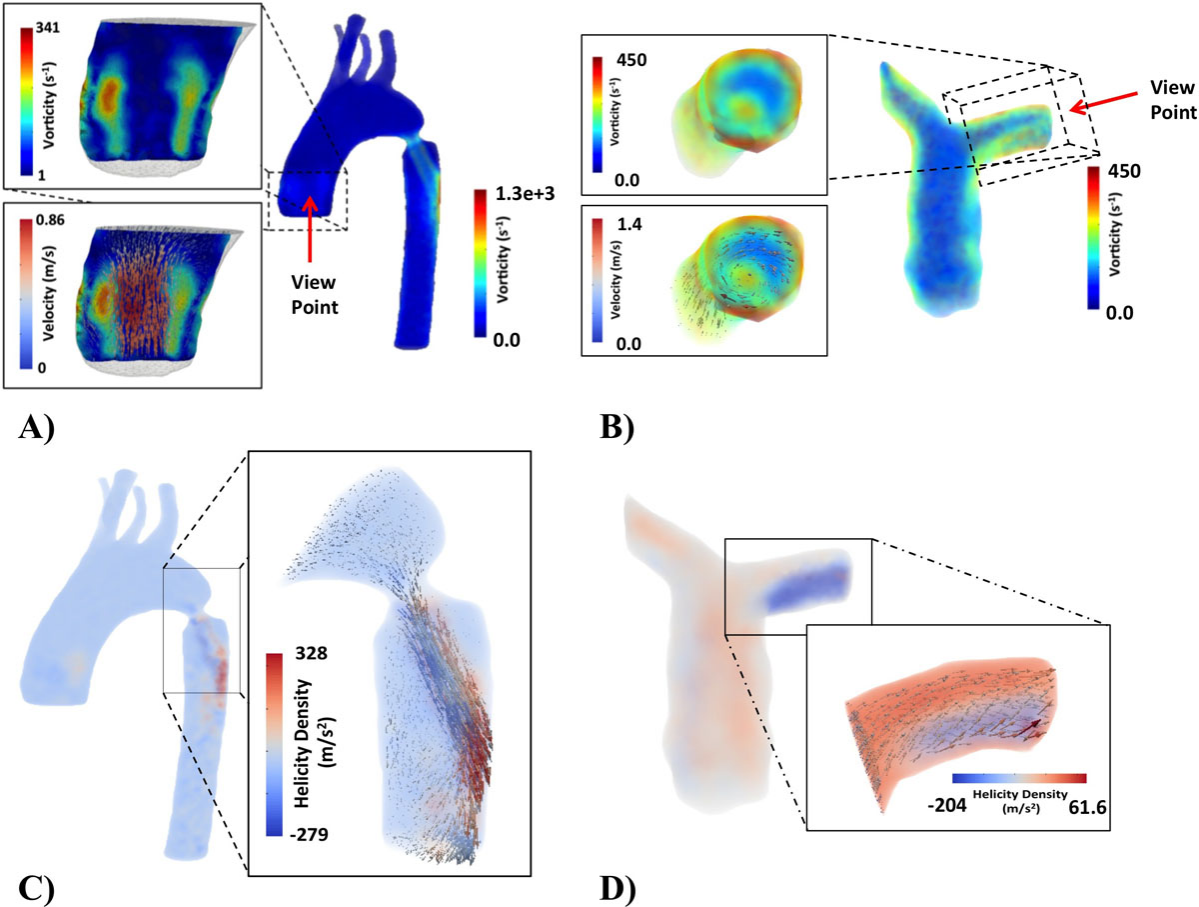
A-B) Vorticity and vector field of velocity, C-D) Helicity Density and vector field of velocity, in the phantom with a coarctation of 9 mm and the pulmonary artery of one volunteer.

The time averaged flow rate from 3D PC-MRI was 61.01±30.42ml/s for the groups of volunteers and 75.71ml//s, 117.55ml//s and 40.80ml//s for the phantom (rest, stress, coarctation) (Fig. 1). 3D maps distribution of WSS and OSI obtain in one volunteer and in the phantom data is shown in Fig. 1. One example of 3D maps of vorticity for the phantom (coarctation) and PA of one volunteer is showing in the Fig. 2. For the Aortic phantom we obtain Vorticity values between 0 to 1300s^-1^, for the PA in volunteers we obtain values between 0 to 450s^-1^. The quantification of the Helicity in 3D domain was applied in the phantom (coarctation), and the result was between -279 to 328m/s^2^ and for the PA of volunteers we obtained Helicity values between -204 to 86m/s^2^.

## Conclusions

We have developed a novel methodology to calculate 3D hemodynamics parameters of a vessel of interest using a combination of 3D cine PC-MRI and finite element interpolations.

## Funding

VRI # 44/2011 (Pontificia Universidad Católica de Chile), Anillo ACT 079 and FONDECYT #11100427 and #11121224. JS thanks CONICYT and Ministry of Education of Chile, with his higher education program, for scholarship for doctoral studies.

